# Sulfur Nutrition Affects Garlic Bulb Yield and Allicin Concentration

**DOI:** 10.3390/plants11192571

**Published:** 2022-09-29

**Authors:** Binh Thi Nguyen, Stephen M. Harper, Tim J. O’Hare, Neal W. Menzies, Bernhard Wehr

**Affiliations:** 1 School of Agriculture and Food Sciences, The University of Queensland, St. Lucia, QLD 4072, Australia; 2 National Institute of Medicinal Materials, Hanoi 134000, Vietnam; 3 The Queensland Alliance for Agriculture and Food Innovation, The University of Queensland, St. Lucia, QLD 4072, Australia

**Keywords:** allium, bulb weight, solution culture, biomass

## Abstract

Improving bulb yield and allicin content of garlic is important in meeting fresh and pharmaceutical market demands. Garlic plants have a high demand for sulfur (S) since allicin contains S atoms. Two experiments were conducted to identify the effect of S application rate on garlic yield and quality. In a field trial assessing six S application rates (0–150 kg S ha^−1^), cultivar ‘Glenlarge’ produced the greatest bulb weight (~90 g) and allicin content (521 mg bulb^−1^) with the application of 75 kg S ha^−1^. In contrast, cultivar ‘Southern Glen’ showed no response in bulb weight or allicin. This was likely due to high soil background S concentrations masking treatment effects. Subsequently, a solution culture experiment with cv. ‘Glenlarge’ evaluated six S application rates (188 to 1504 mg S plant^−1^, nominally equivalent to 25–200 kg S ha^−1^). In solution culture, bulb weight and allicin concentration increased with S rate. Highest bulb weight (~53 g bulb^−1^) and allicin concentration (~11 mg g^−1^ DW) were recorded at an S application of 1504 mg S plant^−1^. This is the first report to conclusively demonstrate the effect of S on yield and allicin in garlic grown in solution culture.

## 1. Introduction

Garlic has been used since ancient times for both culinary and medicinal purposes. Allicin is the main medicinal compound in garlic and a target concentration of >4.5 mg allicin g**^−^**^1^ fresh weight (FW) is preferred by the pharmaceutical industry [1]. Alliin and its derivative allicin give garlic its unique flavour [2] and health benefits. The health benefits of garlic are manyfold, including antimicrobial properties [3] antioxidant activity [4] and anticancer activity [5]. Based on these identified health benefits, numerous commercial products of medicinal value have been made from garlic (e.g.**,** garlic oil, garlic powder and garlic capsules).

Sulfur (S) is a structural element of the allicin molecule and its precursor alliin [2]. The chemical formula of alliin (C_6_H_11_NO_3_S) was first determined by Stoll and Seebeck [6]. Two alliin molecules react to form allicin (C_6_H_10_OS_2_), which contains two S atoms, suggesting that crop S nutrition plays a critical role in contributing to the allicin content of garlic bulbs. The S concentration in garlic bulbs ranges from ~7.4 to 16 g kg^−1^ (dry weight basis (DW) [7]. This value is consistent with other high S demanding crops such as cabbage (~6.6 g kg^−1^) and onion (~5–10 g kg^−1^) [8] and considerably greater than that in tissue of many other plant species, such as wheat (~1.2 g kg^−1^) [9], beetroot (~1.8–3.0 g kg^−1^) and lettuce (3.0 g kg^−1^) [8]. Although garlic has a greater S requirement than many other crops, the S demand is still lower than that for N or K (~20–50 g kg^−1^) [10].

A number of studies have evaluated the effect of S application rate on garlic bulb weight, alliin and allicin concentration, under either field or glasshouse conditions [11,12,13,14]. At S application rates ranging from 0 to 75 kg ha^−1^, bulb weight and allicin concentration was either unaffected [11,12] or increased [14,15]. In these studies, maximum bulb weight was low and ranged from only 8 to 32 g bulb^−1^ [11,14,15]. The highest allicin concentrations across these studies ranged from 5 mg g^−1^ DW [15] to 10.7 mg g^−1^ DW [14] and were lower than those reported in other studies (ranging from ~5.03 to 18.24 mg g^−1^ DW) [16,17]. The lower bulb weights and allicin concentrations reported in these previous studies suggest that S application rates or other growing conditions were not optimal for garlic. Therefore, a field experiment was conducted to evaluate the effect of a range of S application rates on the bulb weight and allicin concentration of two subtropical garlic cultivars, of which cv. Glenlarge is the dominant subtropical variety in Australia.

In soil, S availability and supply can be increased by S fertilizer application (e.g., ammonium sulfate, magnesium sulfate, gypsum or single super phosphate). Furthermore, S may also be derived from atmospheric inputs including air pollution and sea-born sulfate aerosols [17], and soil microbial S cycling [18,19]. Therefore, field experimentation is likely to be less precise in delineating the effect of S application rate on garlic bulb weight and allicin concentration. Hence, a carefully controlled solution culture experiment was conducted to evaluate the effect of S application rate on bulb weight and allicin concentration in garlic bulbs of cv. Glenlarge.

This research aimed to determine how S supply impacts yield and quality of two Australian subtropical garlic cultivars grown in the field and to identify whether solution culture technique can discern S limitations in garlic.

## 2. Results

### 2.1. Effect of Sulfur Application Rates on Garlic Bulb Weight

**Figure 1:**
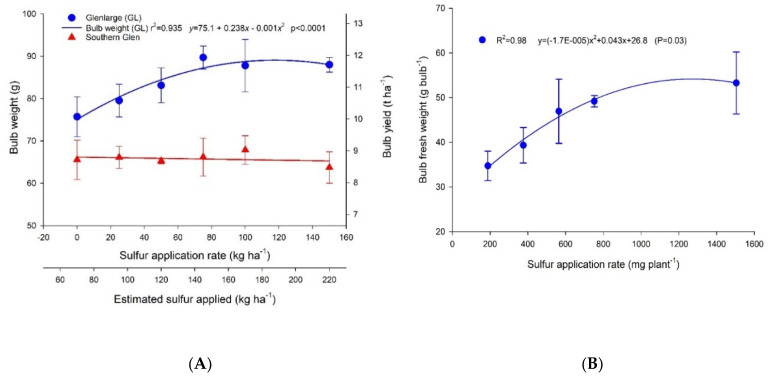
Bulb fresh weight and yield for cultivars Glenlarge (blue lines and symbols) and Southern Glen (red lines and symbols) grown in the field (**A**) and cultivar Glenlarge grown in solution culture (**B**). Values are means of 4 replicates with standard error bars shown if not obscured by the symbols. The lines represent the best-fit linear or quadratic equations.

In the field experiment, a curvilinear regression model was fitted for bulb weight and yield over the S application rates for Glenlarge. This function identifies a maximum bulb weight at a range of S application from 75–150 kg S ha^−1^ (Figure 1A). In contrast, there was no significant effect (*p* > 0.05) of S supply on bulb weight and yield for Southern Glen (Figure 1A). Glenlarge had a greater bulb weight (~90 g bulb^−1^) and fresh yield (~12 t ha^−1^) than Southern Glen (~65 g bulb^−1^ and ~9 t ha^−1^ (fresh yield)) at the optimum S rate of 75 k ha^−1^ (Figure 1A). The estimated sulfur application rate shown in Figure 1A was calculated as the sum of S applied as fertilizer and S applied in irrigation water.

In support of the field trial results, in solution culture the bulb fresh weight of Glenlarge also increased in a curvilinear manner with S application rate (*p* < 0.05) (Figure 1B). Bulb weight was ~35 g bulb^−1^ at an application of 188 mg S plant^−1^, and gradually increased by ~50% to 53 g bulb^−1^ at an application of 1504 mg S plant^−1^. Cultivar Southern Glen was not included in the solution culture trial due to logistical considerations (lack of available space and unsuited to glasshouse temperatures) and time constraints.

The percentage dry matter (DM%) of bulbs grown in the field trial showed no significant difference (*p* > 0.05) when S application rate increased from 0–150 kg ha^−1^, and the DM% was 34% in both garlic cultivars (data not shown). In the solution culture experiment, the DM% of Glenlarge bulbs ranged from ~25–32%, but was not significantly different between S application rates (*p* > 0.05) (data not shown).

### 2.2. Effect of Sulfur Application Rate on S Uptake

The bulb S concentrations showed no significant differences (*p* > 0.05) in the field trial when S application rates increased from 0 to 150 kg ha^−1^ (data not shown). However, the concentration of S (~6.2 g kg^−1^) in bulbs of Glenlarge was lower (*p* < 0.05) than that of Southern Glen (~8.5 g kg^−1^).

**Figure 2:**
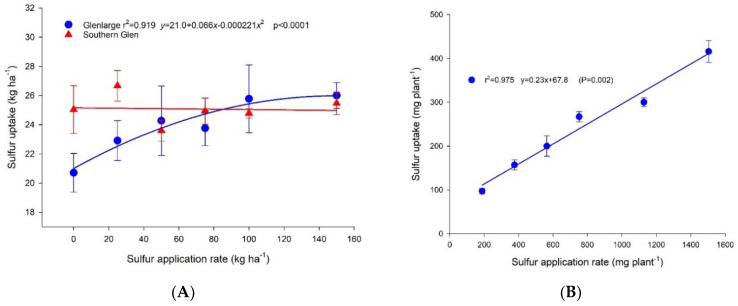
Sulfur uptake for cultivars Glenlarge (blue lines and symbols) and Southern Glen (red lines and symbols) grown in the field (**A**) and cultivar Glenlarge grown in solution culture (**B**). Values are means of 4 replicates with standard error bars shown if not obscured by the symbols.

Sulfur uptake in the field experiment was calculated as the product of S concentration multiplied by bulb dry weight yield. In Glenlarge, the S uptake increased curvilinearly with S application rate, but in Southern Glen, the S uptake was not affected by S application rate (Figure 2A).

In solution culture, S uptake increased linearly from 97 to 416 mg plant^−1^ with increasing S supply from 188 to 1504 mg plant^−1^ (r^2^ = 0.99; *p* < 0.0001) (Figure 2B).

### 2.3. Effect of Sulfur Application Rates on Allicin Concentration and Allicin Content of Garlic Bulbs

Under field conditions, there was a significant difference in bulb allicin concentration and allicin content for Glenlarge (*p* < 0.05) in response to the six S rates (Table 1). Allicin concentration increased by ~20%, from 11.9 to 14.4 mg g^−1^ (DW), when the S application rate increased from 0 to 50 kg ha^−1^. With a further increase in application from 75 to 150 kg S ha^−1^, allicin concentration fluctuated between 13.8 and 14.3 mg g^−1^ DW but the effect was not significant. Bulb allicin content in Glenlarge increased by ~30% when the S application rate increased from 0 to 75 kg ha^−1^ (Table 1). In contrast, in Southern Glen, there was no significant effect of S application rate on allicin concentration (~16.8 mg g^−1^ DW) or bulb allicin content (~375 mg bulb^−1^) (Table 1).

Allicin concentration in Glenlarge (~13.6 mg g^−1^ DW) was substantially lower (*p* < 0.05) than that of Southern Glen (~16.8 mg g^−1^ DW). However, despite this difference, the net allicin content in bulbs of Glenlarge (396 mg bulb^−1^) was significantly greater than that of Southern Glen (375 mg bulb^−1^), across the S application rates, with the exception of the 0 kg S ha^−1^ treatment (Table 1) due to the higher yield potential of Glenlarge.

**Figure 3:**
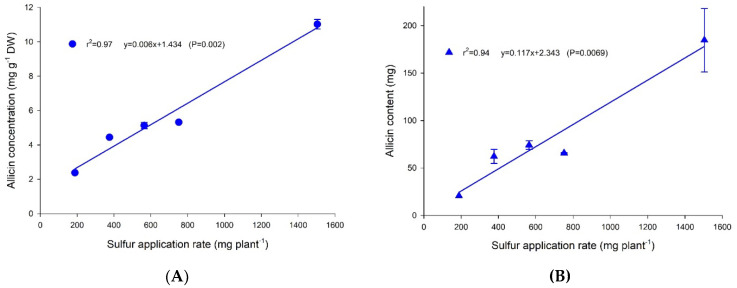
The relationship between sulfur application rate (mg plant^−1^) and (**A**) bulb allicin concentration (mg g^-1^ DW) and (**B**) allicin content (mg bulb^−1^) for garlic cultivar Glenlarge grown in a solution culture at a range of S supply (188–1504 mg plant^−1^). The 1128 mg plant^−1^ treatment was omitted due to bulbs rotting prior to analysis.

The effect of S on allicin was confirmed in solution culture and there were positive linear correlations between S application rate and both allicin concentration and bulb allicin content (Figure 3). Allicin concentration was ~2.3 mg g^−1^ DW at an application rate of 188 mg S plant^−1^ and increased by ~5 times to ~11.0 mg g^−1^ DW at an application rate of 1502 mg S plant^−1^ (Figure 3A) while allicin content increased substantially from ~20 mg bulb^−1^ (at 188 mg S plant^−1^) to ~180 mg bulb^−1^ (at 1504 mg S plant^−1^) (Figure 3B).

## 3. Discussion

### 3.1. Effect of Sulfur Application Rate on Garlic Bulb Weight

In the field trial, across six S application rates, bulb weight of Glenlarge increased by ~20%, from 75.7 to 89.7 g bulb^−1^ (*p* < 0.05) when S application increased from 0 to 75 kg ha^−1^ (Figure 1A), but bulb weight of Southern Glen was not affected (Figure 1B). Despite the low soil mineral S concentration at planting, the irrigation water supplied ~69 kg S ha^−1^ (Table 2) which, together with an unknown supply from soil S mineralisation, may have been sufficient to meet the plant S requirement in the 0 kg ha^−1^ treatment; the estimated S uptake was ~40 kg ha^−1^ for Glenlarge and ~20 kg ha^−1^ for Southern Glen. This would explain why there was no significant difference between the S treatments (not including the 0 kg S ha^−1^ treatment) on garlic bulb weight of Southern Glen under field conditions. Arunachalam et al. [14] conducted an experiment with six S rates ranging from 0 to 75 kg ha^−1^ in three consecutive years and showed that bulb weight increased by ~30%, from ~13 to 17 g bulb^−1^, but the maximum bulb weight was substantially lower than that of the two garlic cultivars grown under field conditions in the present study.

In order to overcome the confounding effect of S supplied through irrigation water, a solution culture experiment was conducted which clearly showed garlic bulb weight increased with S application rate. Garlic bulb weight increased by ~50%, from 35 g bulb^−1^ to 53 g bulb^−1^, when S application increased from 188 to 1504 mg plant^−1^ (nominally equivalent to rates of 25 to 200 kg S ha^−1^ at a plant population of 133,000 garlic plants ha^−1^) (Figure 1B). In the literature, there are no studies about the effect of S application on garlic bulb weight when grown in solution culture, but glass-house pot trials are reported [11,12]. Huchette et al. [8] observed no significant effect of S rates on bulb weight of three garlic cultivars (Printanor, Messidrome and Morasol), which ranged from ~17–23 g bulb^−1^. Losak and Wisniowska-Kielian [12] showed that the bulb weight increased slightly from ~7 to 8 g bulb^−1^ when S was increased from 7 to 22.5 mg plant^−1^. Bulb weights in both these studies were low.

In the present study, the average bulb weight of Glenlarge grown under field conditions (~76–90 g bulb^−1^) (Figure 1A) was substantially higher than that of garlic grown in solution culture (~35–53 g bulb^−1^) (Figure 1B). The lower bulb weight of garlic grown in solution culture might have been due to the higher mean air (18.2 °C) and solution (18.9 °C) temperatures experienced in the glasshouse compared with a mean air temperature of 15.7 °C and mean soil temperature of 14.6 °C under the field conditions (Tables S3 and S4) which may have decreased net CO_2_ assimilation [20]. through increased respiratory losses and heat stress.

### 3.2. Effect of Sulfur Application Rate on S Uptake of Garlic

In the field trial, there were no significant differences in bulb S concentrations across the six S application rates for both Glenlarge and Southern Glen (data not shown). However, Southern Glen bulbs had a higher S concentration (8.5 mg kg^−1^) than Glenlarge (6.2 mg kg^−1^) (*p* < 0.05). The non-significant effect of S application rate on bulb tissue S concentration is perhaps due to the high S supply in the irrigation water, which was estimated at ~69 kg ha^−1^. This supply is greater than the requirement for S in both cultivars and was in excess of S uptake for Glenlarge and Southern Glen in the 0 kg S ha^−1^ treatment. Similarly, the study of Arunachalam et al. [14] showed little effect of S application rates (0–60 kg ha^−1^) on bulb S concentration (~7.0–8.0 mg kg^−1^).

There was a positive curvilinear relationship (*p* < 0.05, r^2^ = 0.919) between S uptake and S application rate for Glenlarge, increasing to ~26 kg ha^−1^ at an application of 150 kg S ha^−1^. However, there was no significant difference in S uptake (~25 kg S ha^−1^) across S application rates for Southern Glen (Figure 2A). This difference might relate to genotypic characteristics in S uptake efficiency or yield potential, with the latter directly determining net S uptake.

In the solution culture experiment, S uptake increased from 97 to 416 mg plant^−1^ (nominally equivalent to 12–55 kg ha^−1^ at a plant population of 133,000 plant ha^−1^) when S application increased from 188 to 1504 (equivalent to 25 to 200 kg S ha^−1^) (Figure 2B). In the lowest solution culture S treatment, despite a limitation of S on growth, a substantial amount of S remained in solution (~50% of the treatment). The S concentration remaining in each treatment solution was positively linearly correlated with S application rate (Figure S2) (*p* < 0.05). This suggests that genetic limitations in S uptake capacity exist in the tested variety Glenlarge.

There were no significant differences in the S or N concentrations in reference leaf samples collected from each S treatment in both the field trial (Table S5) and solution culture (Table S6). Relating the S application rates to bulb yield suggests that maximum bulb yield was obtained when the reference leaf contained > 5.5 mg S kg^−1^ DW and > 45 mg N kg^−1^.

### 3.3. Effect of Sulfur Application Rate on Allicin Concentration and Content in Garlic Bulbs

In the field experiment, S application rates from 25 to 150 kg ha^−1^ did not significantly affect allicin concentration (Table 1). Only Glenlarge showed a 14% increase in allicin when S rate increased from 0 to 25 kg ha^−1^, whereas allicin concentration in Southern Glen (~17 mg g^−1^ DW) showed no S response (Table 1). The allicin concentration in the present study was substantially higher than that in the study of Arunachalam et al. [14]. The solution culture experiment also confirmed that the allicin concentration of Glenlarge increased with S application rate.

The allicin content in bulbs of Glenlarge and Southern Glen responded differently to S application rates due to yield differences across S rates. For Glenlarge, there was a significant increase (*p* < 0.05) in allicin content (~30%), from ~315 to ~420 mg bulb^−1^, when S application increased from 0 to 75 kg ha^−1^ (Table 1). In contrast, the allicin content of Southern Glen (~365–380 mg bulb^−1^) was not significantly different across the six S application rates (*p* > 0.05) (Table 1).

In the solution culture experiment, allicin concentration of Glenlarge increased with S application rate (Figure 3), confirming the results of the field trial. While solution culture allows elucidation of the effect of S rate, plants in solution culture appeared to be more susceptible to postharvest bulb rot. This notwithstanding, there were strong positive linear correlations between S application rate and allicin concentration and content in garlic bulbs.

## 4. Materials and Methods

### 4.1. Field Evaluation of Sulfur Application Rate on Garlic Yield and Allicin

#### 4.1.1. Trial Set-Up

The field experiment was conducted at the Queensland Government Department of Agriculture, Gatton Research Facility (27.56° S, 152.28° E) to evaluate the effect of S application rate on garlic bulb yield and allicin concentration. The soil was a classified as a Brown Dermosol according to the Australian Soil Classification and the soil had pH 7.9, a cation exchange capacity of 27.7 cmol_-c_ kg^−1^ and 7 mg kg^−1^ available sulfur (Table 3). 

Two cultivars were assessed, viz. Glenlarge (high yielding with an intermediate allicin concentration) and Southern Glen (high yielding with a relatively high allicin concentration). Vegetative seed-cloves of these cultivars were selected and planted in a split-plot randomised complete block factorial with 4 replications. The first factor (main plot) was S rate, and the sub-plots were cultivars. The main plots were 4.0 m × 3.0 m, with a 1 m buffer between them. The main plots consisted of two beds of 1.5 m width, with each bed containing 2 rows of plants at 0.55 m spacing. The within-row plant spacing was 10 cm, giving a population density of ~133,000 plants ha^−1^ (Figure S1).

#### 4.1.2. Treatments

Sulfur was added at six rates (0, 25, 50, 75, 100 and 150 kg S ha^−1^) in the form of magnesium sulfate heptahydrate (Table S1). Considering an effective garlic rooting depth of ~30 cm, a soil bulk density of 1.15 t m^−3^, and an exchangeable soil Mg concentration of 12.6 cmol_-c_ kg^−1^, the potential Mg availability in the soil is 200–300 times greater than garlic crop Mg demand (*viz.* 10–20 kg ha^−1^). Hence, we do not consider that the added Mg confounded the S response. Other nutrients were applied at sufficient rates to optimise garlic growth (rates as kg per ha): 180 N, 120 K, 0.7 B, 0.7 Zn, 0.5 Mn, 0.3 Cu, and 0.11 Mo. No P, Ca and Fe was applied because the soil was inherently very high in these nutrients.

The trial was irrigated using Rivulis T-Tape™ 510-20-500 drip irrigation tape with emitter spacing at 20 cm and an output rate of 1 L h^−1^ per emitter. One line of drip tape was installed on the inside of each row of garlic. Fertilizer was applied through fertigation using a partial flow bypass fertigation tank. Irrigation and pesticide applications were made on an as required basis. Pre-plant soil samples (0–15 cm) were collected and a mineral nutrient analysis was conducted (Table 3).

Irrigation water was drawn from an aquifer, and water samples were intermittently collected and analysed for water quality. Samples were stored at −20 °C prior to analysis for N (NO_3_^−^ and NH_4_^+^), K, Na, Ca, Zn, Mg, Mn, Cu, Fe, P, S and B (Table 2). The crop was irrigated at a rate of 4.1 ML per ha and the nutrients supplied by the irrigation water were calculated on a kg per ha basis (Table 2).

#### 4.1.3. Plant Sample Collection

At the flag leaf stage (about 105 days after planting), a composite reference leaf sample (youngest fully expanded leaf) was collected from each replicate of each treatment. The leaf samples were dried at 70 °C for 24 h and analysed for mineral nutrients. At 185 days after planting, the bulbs in each plot were harvested and cured for 28 days in an open-sided well-ventilated shed. Five bulbs (~45–65 mm diameter) of each treatment were selected and used for mineral nutrient and allicin analysis. The samples for allicin analysis included 4 cloves removed from the outer clove-ring of each bulb. The remaining cloves from the bulb were sliced into 3 cm pieces, weighed, dried at 70 °C for 7 days. The dried leaf and bulb samples were sent to Incitec-Pivot (Melbourne, Australia) for mineral nutrient analysis (N, P, K, Ca, Mg, S, Fe, Mn, Zn and Cu). The quantification of allicin was using the method described by Nguyen et al. [21].

### 4.2. Solution Culture Establishment

#### 4.2.1. Trial Set-Up

The solution culture experiment was conducted in a glasshouse at the University of Queensland Gatton Campus (27.56° S, 152.28° E) in 2020 to evaluate the effect of S supply on the growth of garlic and its allicin concentration.

**Figure 4:**
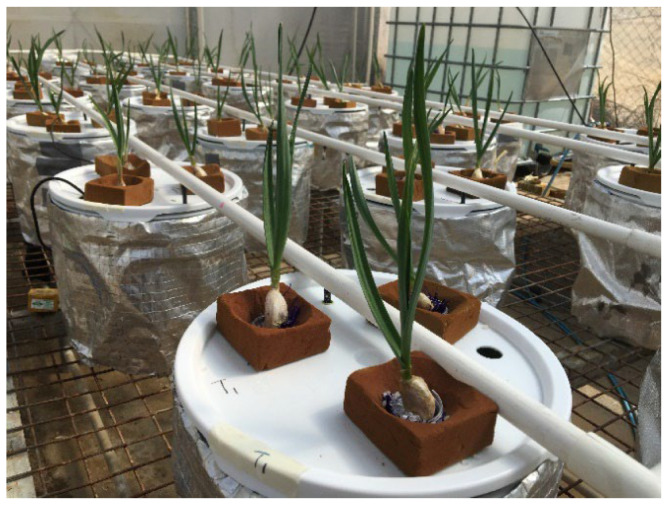
Garlic cloves were grown in solution culture in a glasshouse at Gatton campus, The University of Queensland, Australia in 2020.

Twenty-four 13 L white polythene buckets were filled with 12 L of nutrient solution prepared from aliquots of stock solutions (Table S2). A sleeve of silver-sided Sisalation™ building insulation paper was wrapped around each bucket to moderate solution temperature fluctuations by reducing the effect of direct sunlight on the buckets. The solution in each bucket was aerated using a Resun™ LP100 aquarium air pump with air-lines inserted to each bucket. A plastic lid was prepared with three holes of 50 mm diameter, spaced 10 cm apart to support the plants, and two holes of about 20 mm to accommodate the air-line and to facilitate monitoring of solution pH (Figure 4).

The experiment was conducted on garlic cv. Glenlarge, which is high yielding and with an intermediate allicin concentration, and is the dominant subtropical variety in Australia. Due to logistical and time constraints, cv Southern Glen could not be tested. Seed-cloves for planting were selected from large bulbs (~130 g fresh weight bulb^−1^) which were broken into cloves and uniform cloves with a weight of 6.5 ± 0.5 g selected.

The seed-cloves were imbibed in wet towels for ~7 days to initiate root development and then one clove was transplanted into each of the three cups in each bucket (Figure 4). The cloves were supported in the cups by a piece of plastic netting which was inserted in the base of a plastic cup (diameter of 5 cm) from which the base was removed. After planting the cloves, white plastic beads were placed over the base of the cloves to support the plant.

#### 4.2.2. Treatments

The experiment evaluated the effect of six S rates (188, 376, 564, 752, 1128 and 1504 mg plant^−1^) on plant growth, and alliin and allicin concentration. The S was applied as (NH_4_)_2_SO_4_ and MgSO_4_ with rates equivalent to 25, 50, 75, 100, 150 and 200 kg S ha^−1^ based on a plant population of 133,000 plants ha^−1^. To achieve optimum garlic growth potential, the amount of nutrient per plant was applied at rates of 2114 mg N, 928 mg K, 514 mg P, 487 mg Ca, 159 mg Mg, 6.7 mg Fe, 1.76 mg B, 2.58 mg Zn, 1.81 mg Mn, 0.42 mg Cu, 0.06 mg Mo, 0.30 mg Co and 0.30 mg Ni (Table S2). Mineral element solubility was estimated using the Phreeqc software (version 3.6.2) to ensure there was no precipitation of minerals in the solutions.

Over the duration of the experiment, solution pH was adjusted daily to 6.0 ± 0.2 using dropwise addition of either 0.1 M KOH or HCl. The temperature conditions (ambient and solution) in the glasshouse were monitored using digital recorders (Tables S3 and S4).

#### 4.2.3. Plant Sample Collection

At maturity (~70% foliage senescence, 134 days after planting), the bulbs were harvested and cured (air dried under cover) for 4 weeks. After curing, the bulbs were weighed, and the number of cloves determined in each bulb. Whole bulbs (three per replicate of each treatment) were taken and allicin was determined using the method described by Nguyen et al. [21]. The remainder of this macerated sample was weighed, oven-dried at 70 °C for 7 days, and the dry mass determined.

### 4.3. Statistical Analysis

Data were analysed using Minitab 18.1 software for Windows (Minitab Inc, State College, PA, USA), using one-way and two-way ANOVA. The differences in measured parameters (bulb weight, allicin concentration, allicin content, S concentration) between treatments were determined with Tukey’s HSD at *p* < 0.05. The graphs were presented as mean and standard errors, and appropriate regression analyses conducted using SigmaPlot 14.0 (Systat Software Inc, San Jose, CA, USA).

## 5. Conclusions

In the field trial, Glenlarge showed maximal bulb weight and allicin content at a S application of 75 kg ha^−1^ whereas Southern Glen showed no optimum S response. However, mineralisation of organic S in soil and supply of S in irrigation water likely confounded the S rate response. Hence, a solution culture method was developed to grow garlic plants under controlled S supply. This showed that Glenlarge bulb weight and allicin content were maximal at an equivalent S rate of 1504 mg g^−1^ nominally equivalent to ~200 kg ha^−1^. In solution culture, garlic had only 50% S uptake efficiency and further study is required to understand the factors determining S uptake.

## Figures and Tables

**Table 1:** Effect of sulfur application rate (0–150 kg ha^−1^) on allicin concentration and allicin content in garlic bulbs of cultivars Glenlarge and Southern Glen grown under field conditions.

S Application Rates (kg ha^−1^)	Allicin Concentration (mg g^−1^ DW)		Allicin Content (mg bulb^−1^)	
	Glenlarge *	Southern Glen **	Glenlarge *	Southern Glen **
0	11.9 b	17.3	315 b	385
25	13.6 a	16.9	401 a	378
50	14.4 a	16.4	400 a	364
75	13.8 a	17.1	421 a	374
100	13.6 a	16.3	413 a	381
150	14.3 a	17.0	422 a	367
Mean ***	13.6 a	16.8 b	396 a	375 a

* For cultivar Glenlarge, values in columns followed by different letters are significantly different at *p* = 0.05; ** Treatment effects were not significant (*p* > 0.05); *** Significant differences (*p* = 0.05) in mean allicin between cultivars are denoted by the different letters.

**Table 2:** Nutrient concentration (mg L^−1^) in irrigation water and the calculated nutrient supplied (kg ha^−1^ year^−1^) by irrigation with approx. 4.1 ML ha^−1^ under field conditions.

Mineral	Concentration (mg L^−1^)	Rate Supplied (kg ha^−1^)
NH4^+^	0.05 ± 0.015	0.2
NO3^-^	0.02 ± 0.002	0.08
K	5.0 ± 0.1	20.0
Ca	42 ± 5.4	172
S	16.9 ± 0.9	69
Mg	72 ± 4.4	295
Na	106 ± 1.8	434
P	0.02 ± 0.006	0.08
		(g ha^−1^)
B	0.06 ± 0.001	246
Cu	0.002 ± 0.0003	8.0
Fe	0.003 ± 0.0003	12
Mn	0.003 ± 0.0	12
Zn	0.002 ± 0.0006	8.0

**Table 3:** Soil characteristics at the commencement of the field trial.

Soil Properties	Initial Value (mean ± SD)
Soil pH (1:5 water)	7.92 ± 0.01
EC1:5 (dS m^−1^)	0.14 ± 0.006
Org. C (%) (Walkley-Black)	1.13 ± 0.02
N-NO_3_ (mg kg^−1^)	11.7 ± 2.5
P (mg kg^−1^) (Colwell)	153 ± 5
Cl (mg kg^−1^)	33.0 ± 8.5
CEC (cmol_-c_ kg^−1^) (summation)	27.7 ± 0.6
Ca (cmol_-c_ kg^−1^) (NH_4_-acetate)	12.6 ± 0.35
Mg (cmol_-c_ kg^−1^) (NH_4_-acetate)	12.6 ± 0.57
K (cmol_-c_ kg^−1^) (NH_4_-acetate)	1.20 ± 0.06
Na (cmol_-c_ kg^−1^) (NH_4_-acetate)	0.85 ± 0.06
S (mg kg^−1^) (MCP)	7.0 ± 0.0
Fe (mg kg^−1^) (DTPA)	17.7 ± 1.7
Cu (mg kg^−1^) (DTPA)	4.0 ± 0.4
Zn (mg kg^−1^) (DTPA)	2.1 ± 0.3
Mn (mg kg^−1^) (DTPA)	31.9 ± 5.2

## Data Availability

Not applicable.

## Supplementary Materials

The following supporting information can be downloaded at: https://www.mdpi.com/article/10.3390/plants11192571/s1, Figure S1: Diagram of the experiment plot layout in the field trial in 2020 with two garlic varieties Glenlarge (GL) and Southern Glen (SG); Figure S2: The relationship between S application rate (mg plant^−1^) and sulfur concentration remaining (µM) in solution culture; Table S1: Timing and amount of sulfur (kg ha-1) applied as MgSO_4_ in the field experiment; Table S2: Aliquots of nutrient stock solutions applied to solution culture pots (containing 3 plants each) to meet plant growth demand in an experiment evaluating S rates; Table S3: Soil and solution temperature measured in the glasshouse solution culture experiment and the field experiment conducted in Gatton in 2020; Table S4: Air temperature measured in the glasshouse experiment and the field experiment conducted in Gatton in 2020; Table S5: Effect of S application rate on S, N and C concentration and C:N ratio in the youngest fully expanded leaf of variety Glenlarge growing under field conditions at Gatton Research Facility in 2020.
