# Supplementary material for: Sulfur Nutrition Affects Garlic Bulb Yield and Allicin Concentration
Source: Plants (Basel). 2022 Sep 29;11(19):2571. doi: 10.3390/plants11192571 (PMC9572700; doi:10.3390/plants11192571)
Supplement: Supplementary file 1 [file plants-11-02571-s001.zip › plants-1932797-supplementary.pdf]

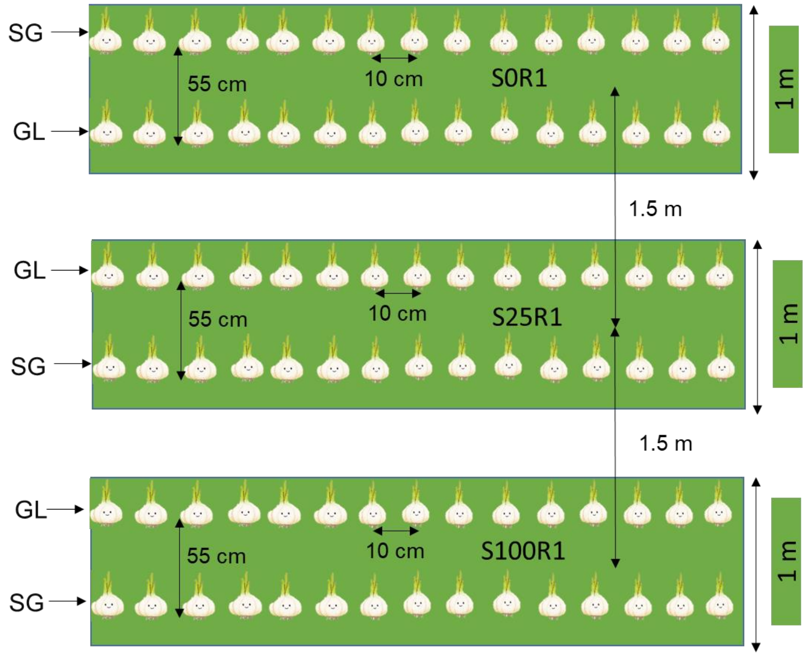

Figure S1. Diagram of the experiment plot layout in the field trial in 2020 with two garlic varieties Glenlarge (GL) and Southern Glen (SG). S denotes sulfur treatment and R denotes replicate.

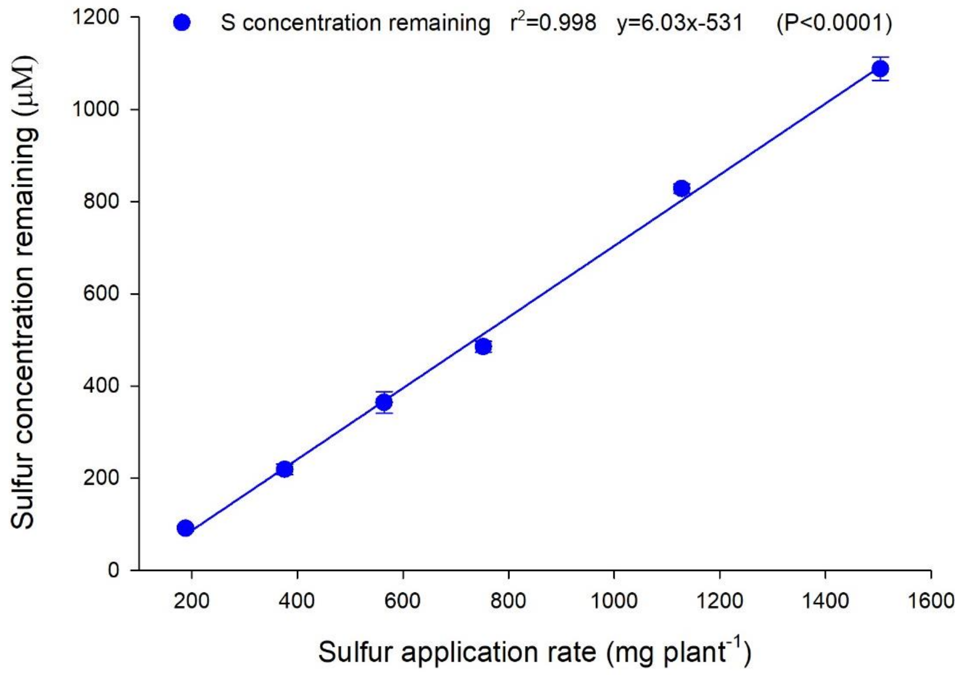

Figure S2 The relationship between S application rate (mg plant^{-1}) and sulfur concentration remaining (μM) in solution culture.

Table S1. Timing and amount of sulfur (kg ha^{-1}) applied as $MgSO_4$ in the field experiment.

S rate (kg ha^{-1})	$MgSO_4.7H_2O$ (kg ha^{-1})	Timing of application (days after planting)		
		10	30	60
0	0	0	0	0
25	609	609	0	0
50	1218	609	609	0
75	1826	609	609	609
100	2434	609	1218	609

150	3652	1218	1218	1218

Table S2. Aliquots of nutrient stock solutions applied to solution culture pots (containing 3 plants each) to meet plant growth demand in an experiment evaluating S rates.

**Fertiliser**	**Stock solution concentration ($g\ L^{-1}$)**	**Weekly (WK) aliquots of stock solution added to pots (ml)**															
		WK 1	WK 2	WK 3	WK 4	WK 5	WK 6	WK 7	WK 8	WK 9	WK 10	WK 11	WK 12	WK 13	WK 14	WK 15	WK 16
**All pot applications**																	
$(NH_4)_2SO_4$	20.3	6	8	0	8	4	0	0	4	0	0	0	0	0	0	-	-
$(NH4)NO_3$	230.3	12	16	0	5	8	8	0	4	0	4	0	0	0	0	-	-
$Ca(NO_3)_2.4H_2O$	126.3	6	8	9	0	8	8	2	9	0	4	8	0	4	0	-	4
KNO_3	133.8	6	8	5	0	4	5	0		2	0	4	0	0	0	-	-
KH_2PO_4	74.3	6	8	4	0	4	5	9	4	0	8	0	0	0	0	-	5
$MgCl_2.6H_2O$	62.7	3	4	2	1	2	2	2	2	2	2	0	0	0	0	-	-
$MgSO_4.7H_2O$	57.2	8	12	0	8	4	-	-	4	4	8	4	3	0	-	-	-
$CaCl_2$	2.1	12	16	9	0	5	2	2	0	0	0	0	0	0	0	-	-
$CaCl_2$	4.3	0	0	0	-	-	-	-	2	-	4	-	-	-	-	-	-
Sodium-FeEDTA	2.45	4	4	4	-	4	4	8	4	-	6	4	8	-	4	-	4
H_3BO_3	0.76	4	4	4	-	8	2	2	2	4	2	2	2	2	-	2	-
$MnCl_2.4H_2O$	0.43	4	4	4	-	4	4	2	2	2	2	2	4	6	2	2	2
$Na_2MoO_4.2H_2O$	0.01	4	4	4	-	12	-	2	2	2	-	2	2	4	-	2	2
$ZnSO_4.7H_2O$	1.06	4	6	2	4	4	-	2	2	2	2	2	2	-	-	-	-
$CuSO_4.5H_2O$	0.08	4	6	2	4	4	-	4	2	6	3	4	13	6	2	2	-
$CoSO_4.7H_2O$	0.68	4	6	-	2	1	-	2	-	2	-	2	-	2	-	2	-
$NiSO_4.6H_2O$	0.20	4	6	-	2	1	-	2	-	2	-	-	-	2	-	-	-

**S Treatment applications $MgSO_4.7H_2O$**																	
T2	216.5	2	2	4	4	2	-	-	2	1	1	1	1	-	-	-	-
T3	216.6	4	4	8	8	4	-	-	4	2	2	2	2	-	-	-	-
T4	216.6	6	6	12	12	6	-	-	6	3	3	3	3	-	-	-	-
T5	216.6	10	10	20	20	10	-	-	10	5	5	5	5	0	-	-	-
T6	216.6	14	14	28	28	14	-	-	14	7	7	7	7	0	-	-	-

Table S3. Soil and solution temperature measured in the glasshouse solution culture experiment and the field experiment conducted in Gatton in 2020. The data includes average daily temperatures expressed on a per week basis for the six-week period from 23 July 2020 to 2 September 2020.

Week	Glasshouse solution temperature (°C)			Field soil temperature (°C)		
	Mean	Maximum	Minimum	Mean	Maximum	Minimum
1	17.9	19.9	16.1	14.6	16.2	13.2
2	18.2	21.2	15.4	14.2	16.9	11.9
3	18.5	21.1	16.0	14.2	16.3	12.3
4	19.8	22.8	16.9	15.0	17.4	12.9
5	18.5	21.9	15.1	13.7	16.6	11.1
6	21.0	24.7	17.4	15.7	18.9	12.9

Table S4. Air temperature measured in the glasshouse experiment and the field experiment conducted in Gatton in 2020. The data includes average daily temperatures expressed on a per week basis for the twelve-week period from 01 May 2020 to 23 July 2020.

Week	Glasshouse air temperature (°C)		Field air temperature (°C)	
	Minimum	Maximum	Minimum	Maximum
1	10.4	29.4	12.2	24.4
2	12.2	29.8	13.8	26.0
3	13.1	28.0	7.9	24.1
4	11.1	25.6	8.6	21.4
5	10.7	27.1	7.7	23.4
6	10.4	27.0	6.0	24.3
7	13.0	26.0	5.9	24.1
8	9.8	23.2	8.7	20.6
9	8.2	24.0	8.3	22.6
10	8.3	24.4	8.3	22.1
11	10.1	23.6	8.5	20.4
12	8.6	23.4	5.0	22.4

Table S5. Effect of S application rate on S, N and C concentration and C:N ratio in the youngest fully expanded leaf of variety Glenlarge growing under field conditions at Gatton Research Facility in 2020.

S treatments ($kg\ ha^{-1}$)	S ($g\ kg^{-1}$)^{NS}	N ($g\ kg^{-1}$)^{NS}	C (%)^{NS}	C:N^{NS}
0	5.48	45.5	44.0	9.8
25	5.72	45.5	43.7	9.7
50	5.58	45.6	44.3	9.6
75	5.76	46.0	43.8	9.5
100	5.54	45.9	44.0	9.6
150	5.37	46.5	43.9	9.3
**Mean**	**5.58**	**45.8**	**43.9**	**9.6**

^{NS} denotes no significant difference between S treatments (P>0.05)

Table S6. Sulfur, N and C concentration and C:N ratio in the youngest fully expanded leaf of garlic variety Glenlarge at different S application rates in solution culture.

S application rate ($mg\ plant^{-1}$)	S ($g\ kg^{-1}$)^{NS}	N ($g\ kg^{-1}$)^{NS}	C%^{NS}	C:N^{NS}
188	6.05	59.9	45.1	7.5
376	5.95	59.8	45.0	7.5
564	5.93	58.9	45.0	7.6
752	6.20	60.4	45.0	7.5
1128	6.25	60.4	45.0	7.5
1504	6.04	60.4	45.0	7.5
**Avg**	**6.07**	**60.0**	**45.0**	**7.5**

^{NS} denotes no significant difference between S treatments (P>0.05)
